# Supplementary figures and images for: Axonal distribution of mitochondria maintains neuronal autophagy during aging via eIF2β
Source: eLife. 2026 Jan 26;13:RP95576. doi: 10.7554/eLife.95576 (PMC12834499; doi:10.7554/eLife.95576)

**Figure 2** Fig. 2A

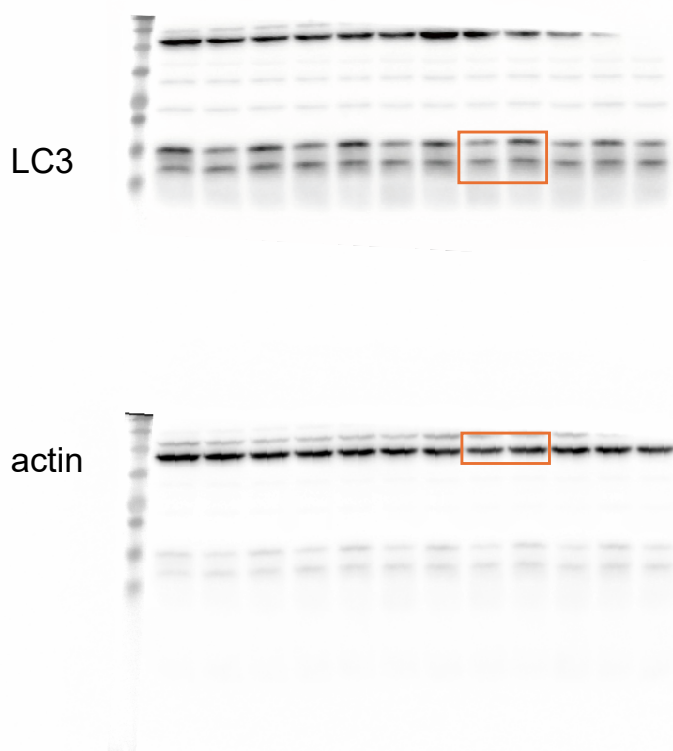

Fig. 2B

p62

actin

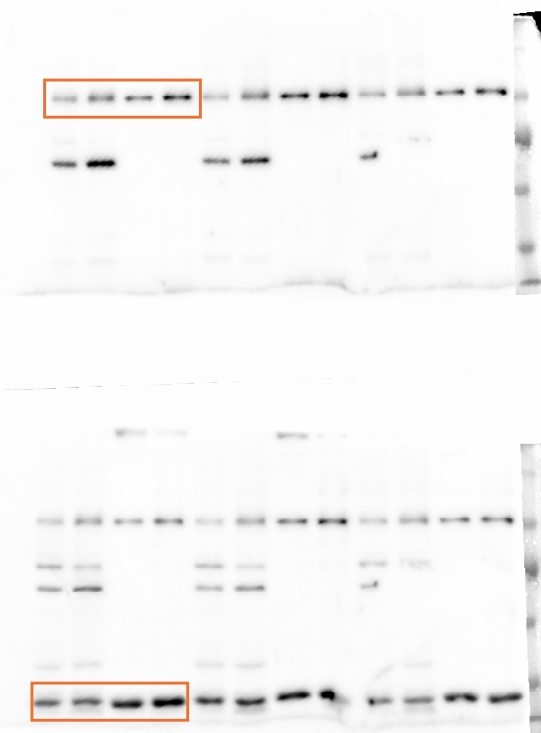

Fig. 2D

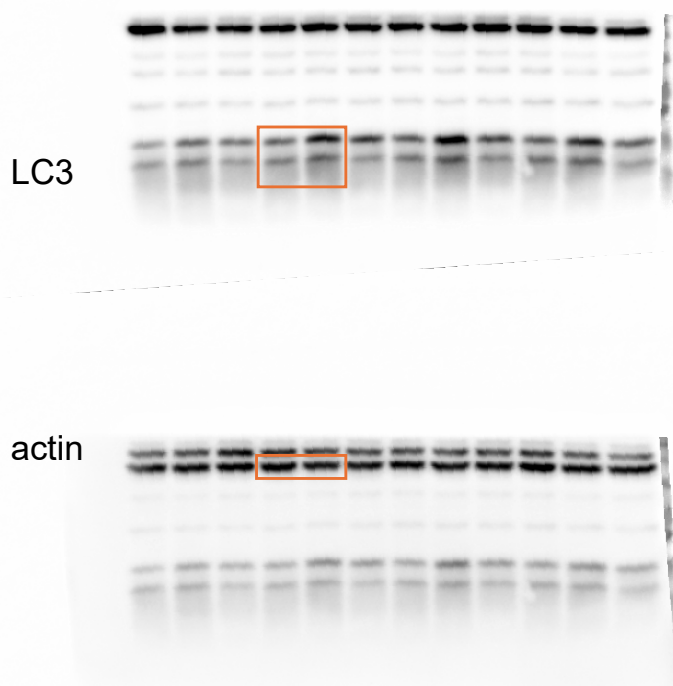

Fig. 2E

p62

actin

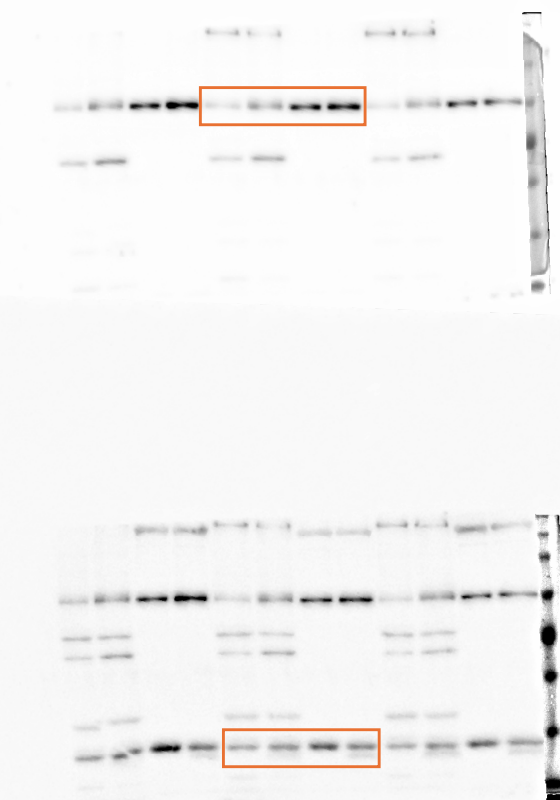

Supplement: Figure 2—source data 1. [file elife-95576-fig2-data1.zip › Figure 2_Source data 1.pdf]

**Figure 3** Fig. 3D

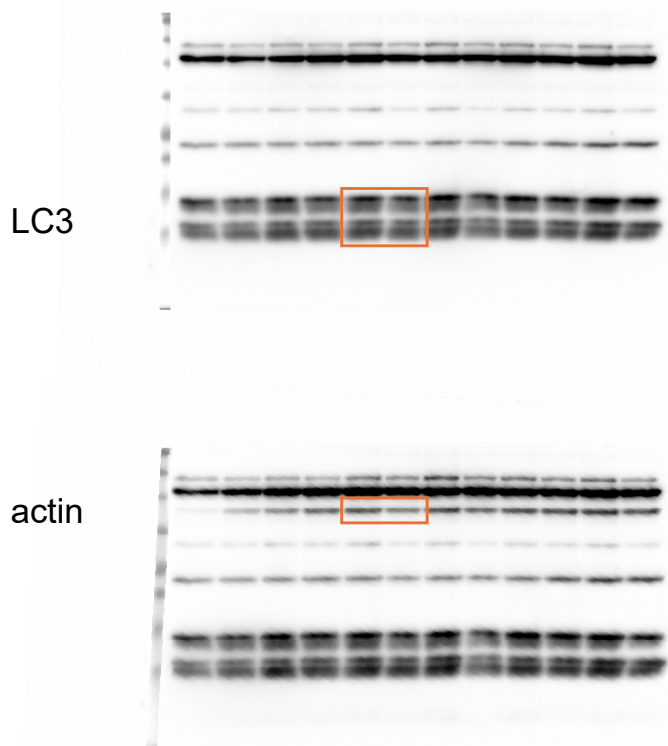

Fig. 3E

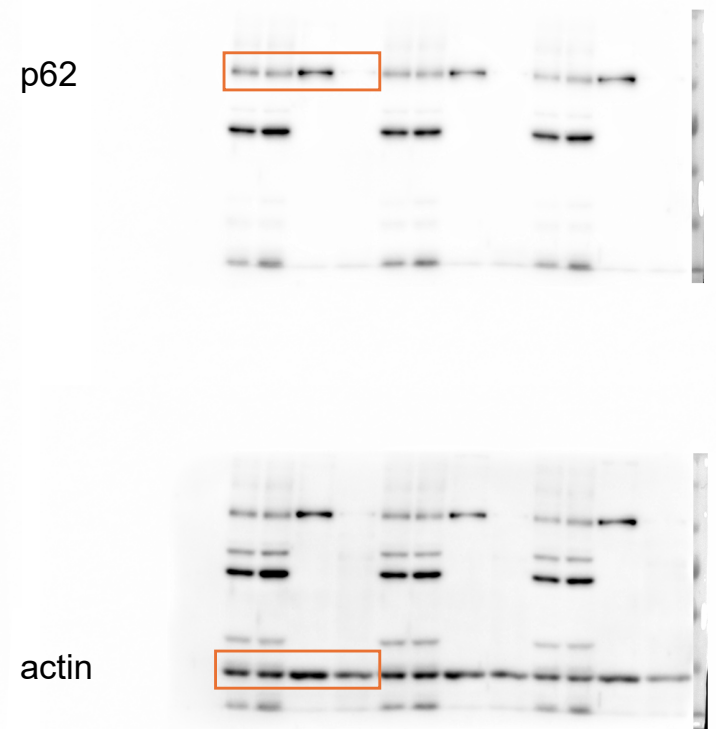

Supplement: Figure 3—source data 1. [file elife-95576-fig3-data1.zip › Figure 3_Source data 1.pdf]

**Figure 4** Fig. 4E

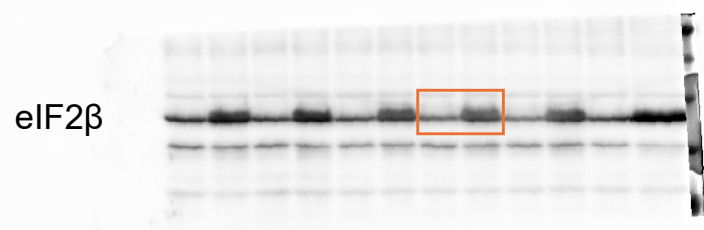

Fig. 4G

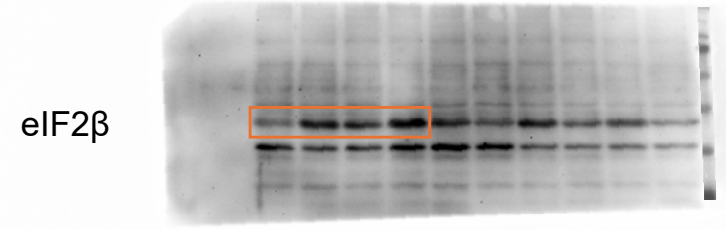

Supplement: Figure 4—source data 1. [file elife-95576-fig4-data1.zip › Figure 4_Source data 1.pdf]

**Figure 5** Fig. 5A

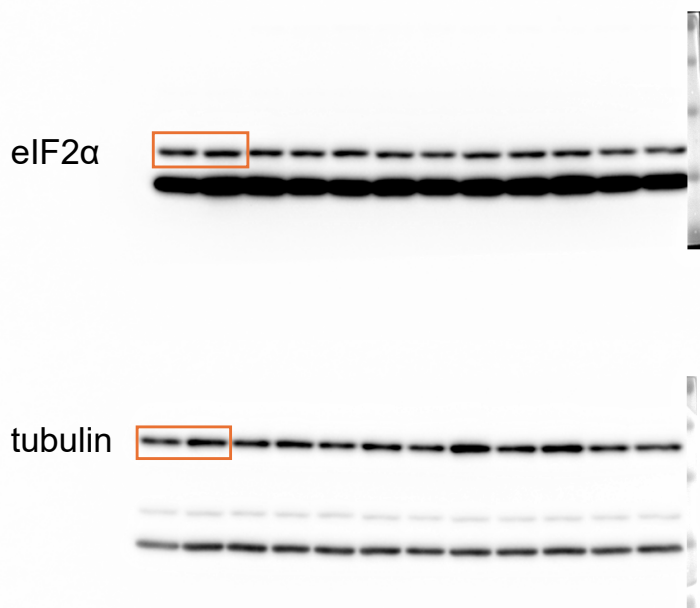

Fig. 5B

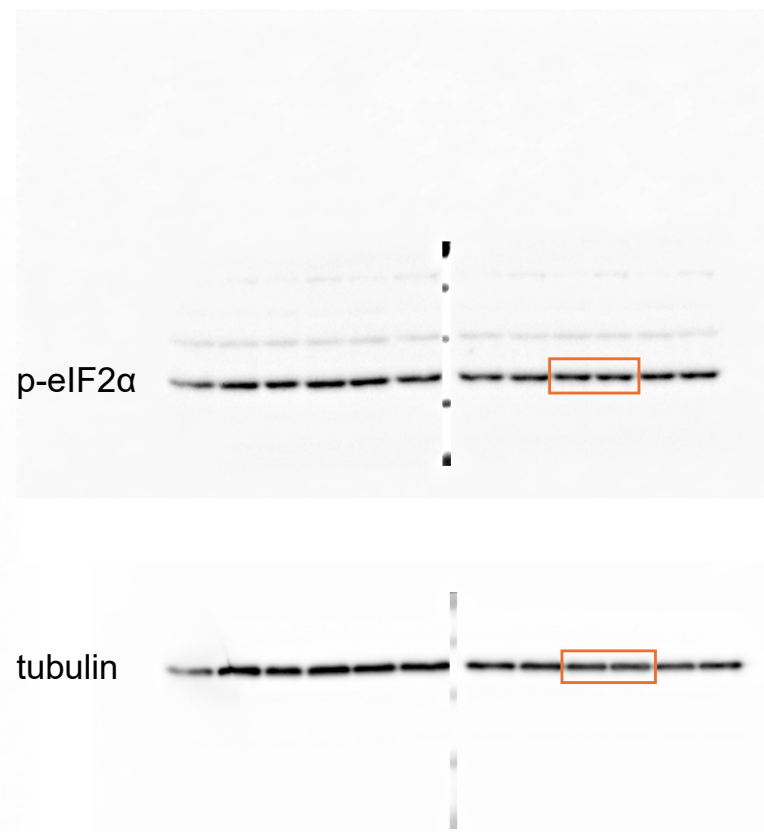

Supplement: Figure 5—source data 1. [file elife-95576-fig5-data1.zip › Figure 5_Source data 1.pdf]

# Figure 6

Fig. 6C

puromycin

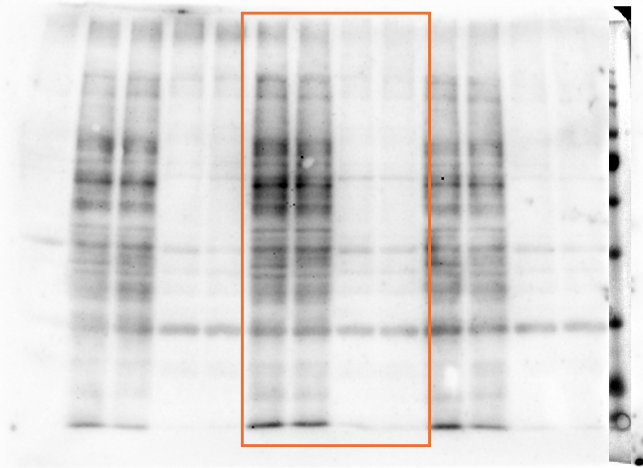

actin

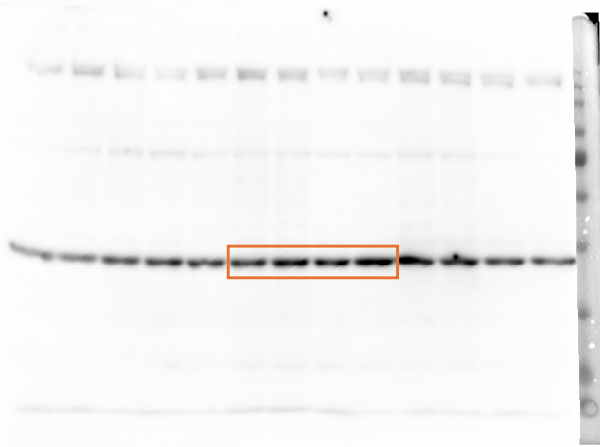

Supplement: Figure 6—source data 1. [file elife-95576-fig6-data1.zip › Figure 6_Source data 1.pdf]

**Figure 7** Fig. 7B

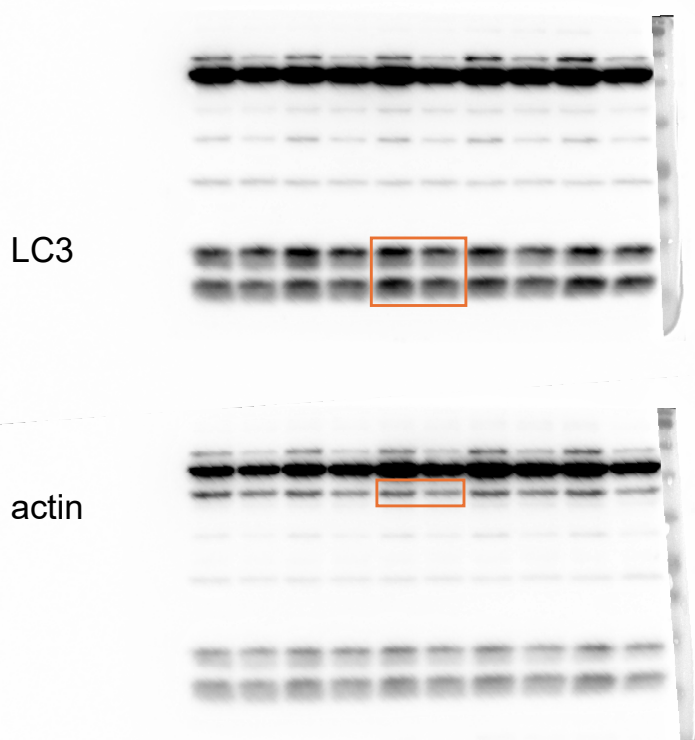

Fig. 7C

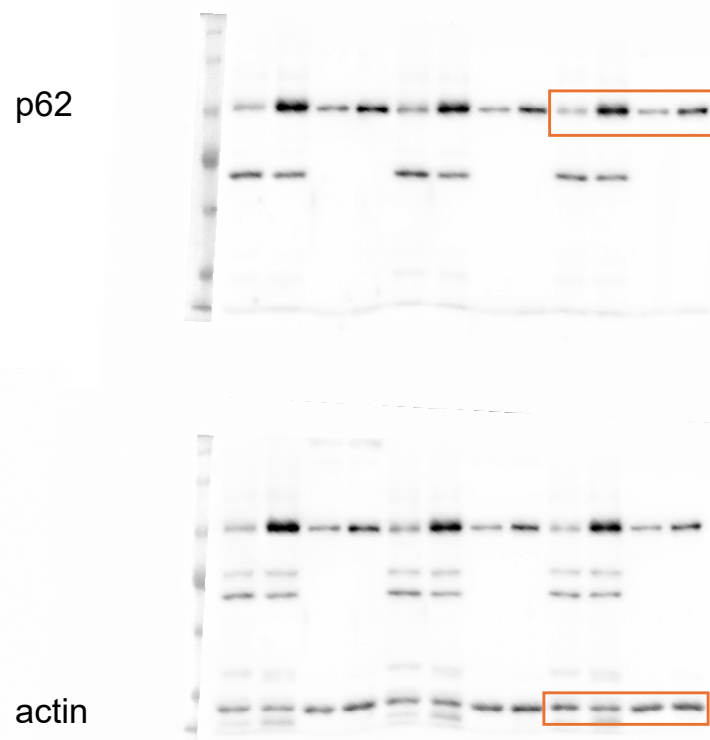

Fig. 7D

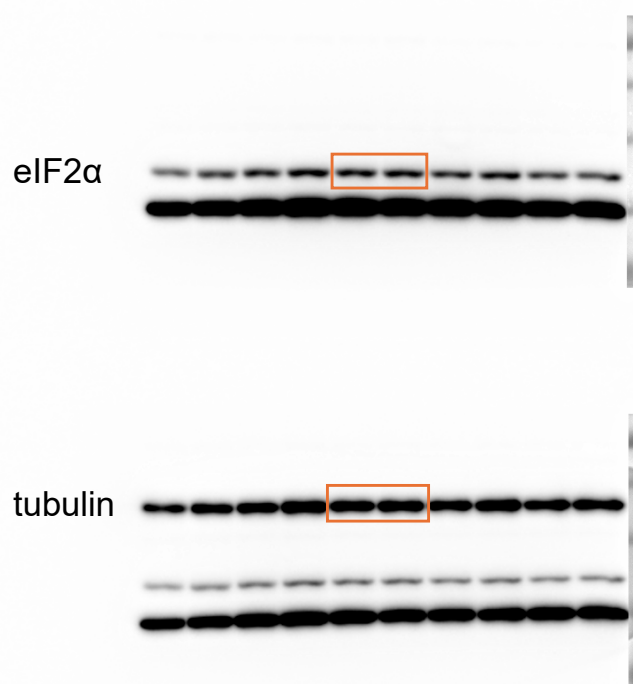

Fig. 7E

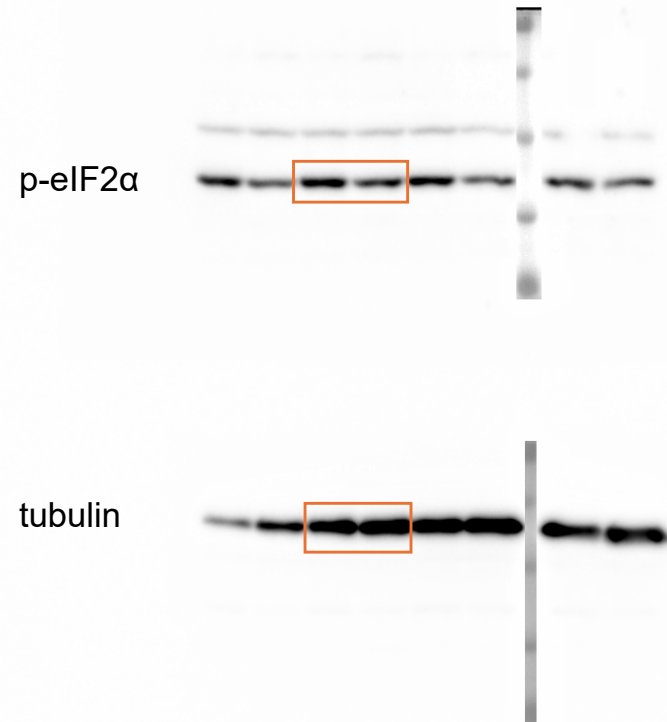

Supplement: Figure 7—source data 1. [file elife-95576-fig7-data1.zip › Figure 7_Source data 1.pdf]

**Figure 8** Fig. 8B

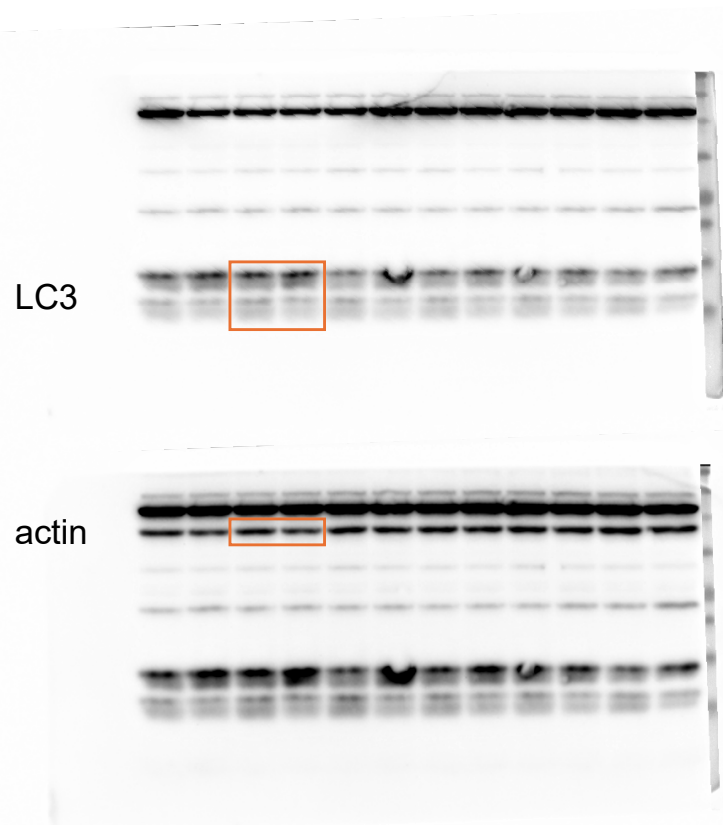

Fig. 8C

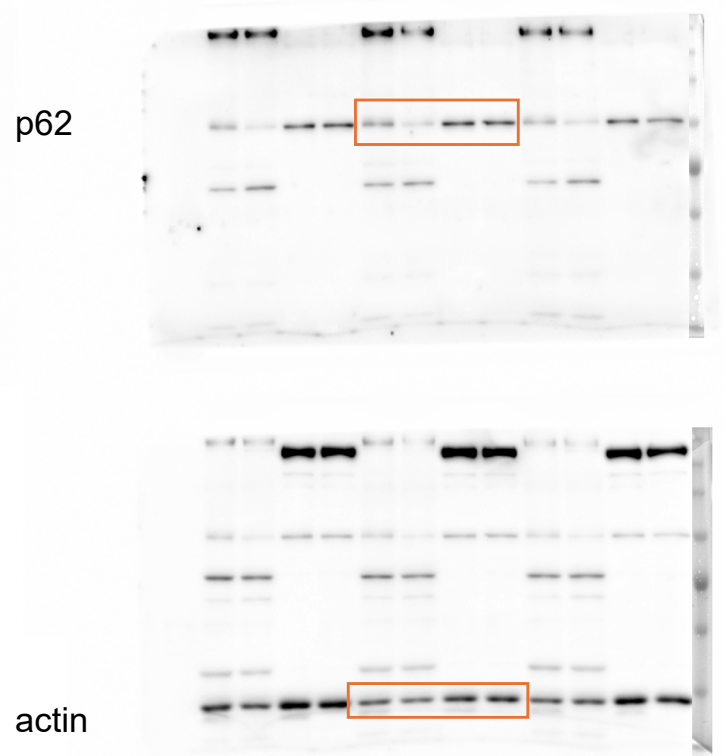

Supplement: Figure 8—source data 1. [file elife-95576-fig8-data1.zip › Figure 8_Source data 1.pdf]

**Figure 8** Fig. 8B

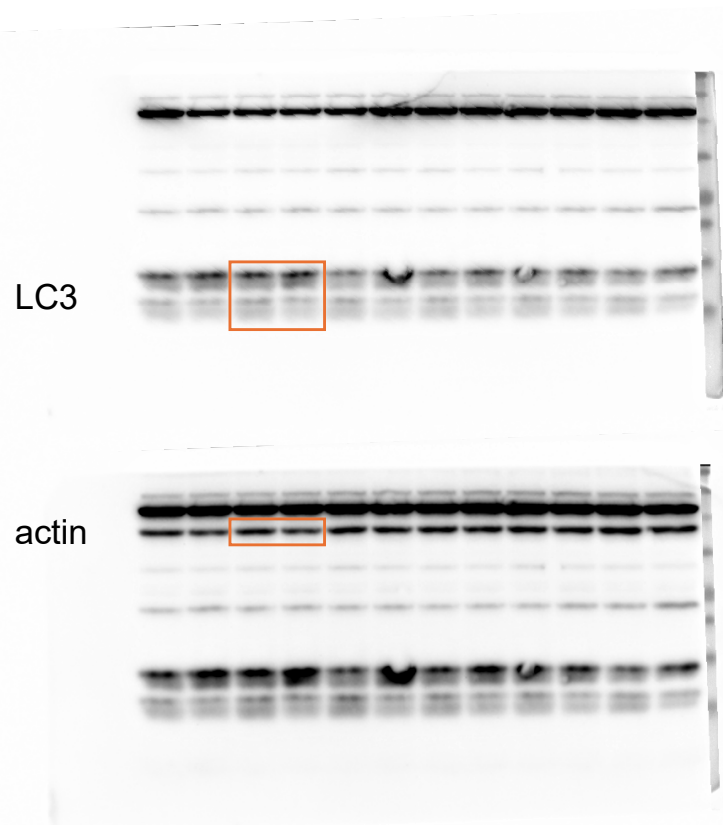

Fig. 8C

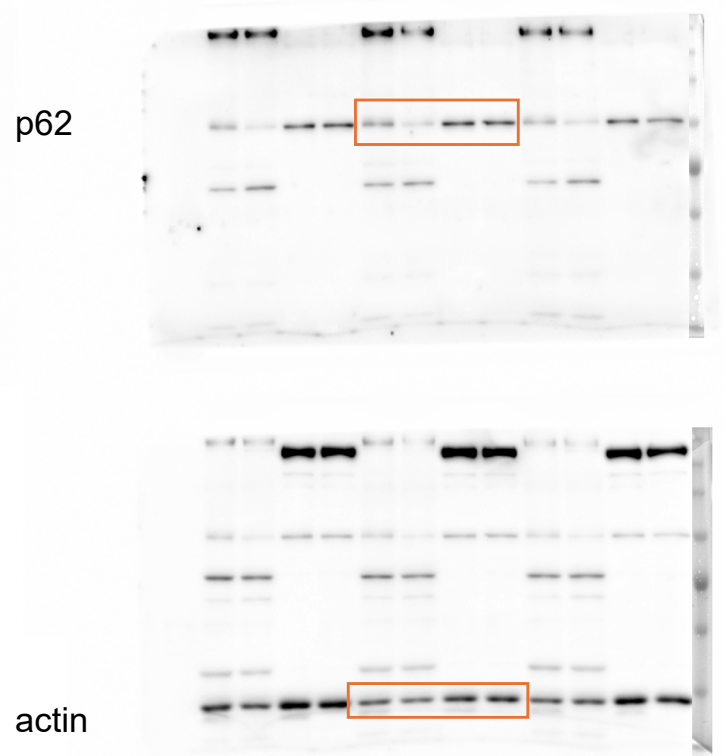

Supplement: Figure 8—figure supplement 1—source data 1. [file elife-95576-fig8-figsupp1-data1.zip › Supplemetal figure 3_Source data 1.pdf]
